# The Yin and the Yang of Hemostasis in End-Stage Liver Disease

**DOI:** 10.3390/jcm12175759

**Published:** 2023-09-04

**Authors:** Fuat H. Saner, Ecaterina Scarlatescu, Dieter Clemens Broering, Dmitri Bezinover

**Affiliations:** 1 King Faisal Specialist Hospital & Research Center, Organ Transplant Center of Excellence, Riyadh 11564, Saudi Arabia; dbroering@kfshrc.edu.sa; 2 Department of Anesthesia and Intensive Care Medicine III, Fundeni Clinical Institute, 022328 Bucharest, Romania; katyscarlatescu@yahoo.com; 3 Anesthesia and Intensive Care Department, University of Medicine and Pharmacy “Carol Davila”, 050474 Bucharest, Romania; 4 Department of Anesthesiology and Critical Care, Hospital of the University of Pennsylvania, Philadelphia, PA 19104, USA; dbezinover@gmail.com

**Keywords:** liver cirrhosis, fibrinolysis, liver and sepsis, hemostasis monitoring

## Abstract

Patients with end-stage liver disease (ESLD) undergoing liver transplantation (LT) are prone to thromboses both while on the waiting list and in the perioperative period. This hypercoagulability is associated with significant endothelial dysfunction (ED) due to nitric oxide dysregulation. ED and increased thrombin generation are the main factors responsible for this hypercoagulability. Sepsis alone can significantly alter a patient’s coagulation profile. In combination with ESLD, however, sepsis or septic shock are responsible for very complex changes. This makes both the assessment and management of coagulation in septic patients with ESLD very challenging. Viscoelastic testing (VET) is the preferred method of coagulation management in patients with cirrhosis because, as with standard laboratory testing, VET can assess the entire coagulation system including the interaction between both pro- and anticoagulants and platelets.

## 1. Introduction

End-stage liver disease (ESLD) is associated with significant changes in a patient’s coagulation profile. These changes are unique in that they involve all branches of the coagulation system. Despite a significantly reduced concentration of coagulation factors and platelets, patients with ESLD may have provoked or unprovoked bleeding or clotting events.

This manuscript discusses some unique aspects of coagulation in cirrhotic patients such as hypercoagulability, thromboses associated with ESLD, and specific subpopulations susceptible to thromboembolism.

We also reviewed the association between sepsis and ESLD. While sepsis itself is associated with changes in coagulation, in combination with hepatic impairment, the entire coagulation profile is significantly altered resulting in unpredictable bleeding or clotting events.

Viscoelastic testing (VET) has been used for many years to manage coagulation in patients with ESLD. In this manuscript, we discussed why VET, rather than standard laboratory testing (SLT), is the preferred method for coagulation management in patients with ESLD.

## 2. Hypercoagulability in End-Stage Liver Disease: Pathophysiology and Susceptible Populations

### 2.1. End-Stage Liver Disease and Venous Thromboembolism 

ESLD can be associated with both severe bleeding and clotting. The majority of coagulation and anticoagulation factors are produced in the liver. Despite a reduction in the production of most of these factors in ESLD and defects in primary, secondary, and tertiary coagulation, the coagulation system is most often stable. Some of the coagulation factors produced are extrahepatic and this is one of the main reasons hepatic failure is not necessarily associated only with spontaneous unprovoked bleeding, but with clotting as well [1]. One of the first publications demonstrating that patients with ESLD are not naturally anticoagulated but prone to venous thromboembolism (VTE) was published in 2009 by Northup et al. [2]. The authors found that the incidence of VTE in hospitalized cirrhotic patients was 0.5%. Most of these patients also had a low serum albumin level (most likely a surrogate marker for circulating anticoagulants). In an evaluation (2009) based on the Danish National Register, Søgaard et al. demonstrated that both patients with cirrhosis and not-cirrhotic chronic liver disease had a high risk of developing VTE (relative risk for thromboses 2.06 and 2.10, respectively) in comparison to matched controls from the general population [3]. An association between chronic liver disease and VTE was later confirmed in several publications with an incidence of between 0.5% and 8% [4,5,6,7]. This wide range likely reflects the variability in published studies related to the population studied, particularly as it relates to severity and etiology of liver disease and the diagnostic criteria used. If patients with ESLD require LT, the concern for perioperative thrombotic or bleeding complications is even higher. In fact, different types of thromboses have been described during each stage of transplant surgery.

### 2.2. Types and Prevalence of Perioperative Thromboembolic Events 

Portal vein thromboses (PVT) are more frequently seen preoperatively with a prevalence of between 1% and 16%, occurring more frequently in patients with decompensated disease [8,9]. An association between preoperative PVT and increased postoperative mortality has been demonstrated [10,11,12]. Like VTE, significant variability in the prevalence of PVT is most likely related to differences in the study populations and design. The vast majority of published studies are single-center and retrospective.

Intraoperative thrombotic complications with clinical presentation occur with a frequency of 1–6% [13,14]. However, transesophageal echocardiography is routinely used during surgery, and a variety of clots have been seen in almost half of all cases, with the majority not needing any treatment [15]. Symptomatic clots, in the form of pulmonary emboli (PE) or intracardiac thromboses, can occur at each stage of LT surgery, but are most frequently described at the time of graft reperfusion and are associated with significant hemodynamic instability and a high mortality rate [16].

Different types of thrombotic complications occur postoperatively. It has been demonstrated that preoperative PVT is associated with postoperative thrombotic complications [10,17,18]. Postoperative thromboses can manifest in the form of PVT (2%) [17], hepatic artery thromboses (HAT) (3–6% and over 8% in children) [19,20], and VTE (5–10%) [21]. Many of these complications are associated with increased mortality. The incidence of PE after LT is about 4% [16] with an associated one-year mortality rate as high as 12% [22]. Early (within 90 days after LT) HAT is associated with graft failure and requires re-transplantation with a rate above 50% for adults and above 60% in children [17,19]. The development of postoperative thromboses (especially HAT) is significantly affected by surgical technique and perioperative management. 

### 2.3. Causes of Thromboembolism in Cirrhosis

Although the cause of hypercoagulability in ESLD is multifactorial, endothelial dysfunction (ED) is most likely the main driving mechanism of clotting in cirrhotic patients. The pressure gradient between the systemic and portal circulation results in increasing intravascular shear forces resulting in activation of nitric oxide (NO) production and the development of ED [23]. Increased NO levels lead to significant vasodilation, primarily in the portal circulation, resulting in a “steal effect” in the systemic circulation. Low systemic pressure is responsible for a dramatic decrease in perfusion of the intestines, which is associated with increased mucosal permeability, release of endotoxins, and secondary activation of the NO pathway, resulting in an even higher degree of systemic hypotension and ED [24]. Other factors contributing to the development of ED in cirrhotic patients are increased inflammatory and oxidative stress [25]. Endotoxemia itself is associated with increased thrombin generation [26], which is one of the major factors in hypercoagulability. ED in patients with ESLD is also associated with resistance to thrombomodulin (TM), a membrane protein expressed on the surface of endothelial cells, that serves as a co-factor for protein C activation with subsequent impairment of the anticoagulation pathway and increase in thrombin generation [27,28]. Additionally, both the hepatic production of proteins C and S, and antithrombin III (ATIII), and their activity are significantly reduced in ESLD [29]. Another ED-related factor contributing to hypercoagulability in cirrhosis is the significantly increased production of endothelium liver-independent coagulation or anticoagulation factors such as Factor (F) FVIII, von Willebrand factor (vWF), and plasminogen activator inhibitor 1 (PAI-1) [30,31,32]. vWF is likely one of the key components of hypercoagulability in cirrhosis. Despite thrombocytopenia and impaired platelet function in vitro [33], platelet function in vivo is likely normal or even increased due to a high concentration of vWF. This is not just because of increased production but also because of decreased destruction by cleaving protease ADAMTS13 synthetized in the liver [29,34]. 

FVIII is one of the targets of activated protein C. In ESLD, the ratio between FVIII and activated protein C (which indicates the severity of cirrhosis) can reach 5.0. This imbalance is an additional factor responsible for the impairment of thrombin generation inhibition [35,36].

**Figure 1:**
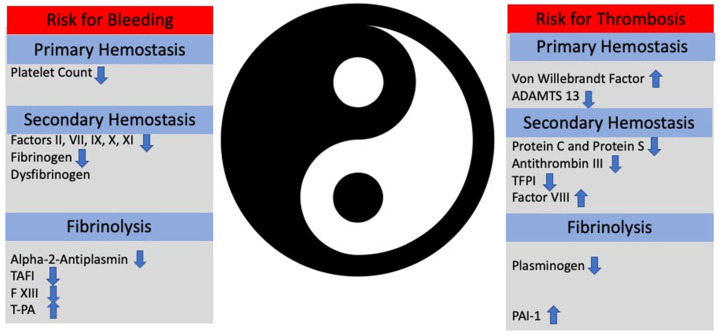
The Ying (bleeding) and the Yang (thrombosis) of hemostasis in patients with end-stage liver disease. Instable balance between thrombosis and bleeding illustrates the critical balance between pro- and anticoagulants. Depending on clinical scenario, patients can bleed or develop thrombosis. TAFI: tissue activable factor inhibitor. t-PA: tissue plasmin activator. PAI-1: plasminogen-activator inhibitor-1.

In addition to primary and secondary coagulation, tertiary coagulation is significantly affected in cirrhosis. An increase in PAI-1 levels in combination with reduced levels of tissue plasminogen activator is responsible for decreased fibrinolysis in patients with ESLD [37,38]. It has been also shown that patients with ESLD have increased clot stability due to diminished permeability and reduced lysis [39,40] (Figure 1).

### 2.4. Subpopulations Susceptible to Thromboembolism

There are number of subpopulations of patients with ESLD predisposed to developing thromboses. It has been demonstrated that patients with nonalcoholic steatohepatitis (NASH) have an increased prevalence of deep vein thrombosis, PE, and PVT [41,42,43]. The reason for this is inflammation and increased oxidative stress that occurs with NASH at a higher degree than in patients with other types of ESLD [37,44]. Other subpopulations prone to thrombotic complications include patients with autoimmune conditions (due to chronic inflammation, cytokine release, and high fibrinogen concentration) [45,46] and patients with hepatitis C (due to chronic inflammation and production of autoantibodies such anticardiolipins and increased thrombin generation) [47,48].

Several genetic mutations associated with thromboses (mostly VTE and PVT) are frequently seen in patients with ESLD. These include FV Leiden [49], Prothrombin G20210A mutation (which leads to a high prothrombin level) [50], high plasma homocysteine level [51], a mutation in the methylenetetrahydrofolate reductase gene 51, JAK2V617F mutations [52], and myeloproliferative neoplasms (MPN) [53]. Due to chronic hypercoagulability associated with ESLD, these mutations make patients with cirrhosis more susceptible to thromboses than the general population.

## 3. Monitoring of Hemostasis in Patients with End-Stage Liver Disease

Although SLTs have never been formally evaluated for predicting bleeding or for treatment, these tests are mainly used for managing patients with severe trauma, cardiac surgery, and liver-related hemostasis disorders. Tests such as the prothrombin time (PT), activated partial thrombin time (aPTT), international normalized ratio (INR), fibrinogen, and platelet count have significant limitations with regard to evaluating clot formation and kinetics as well as clot strength [54]. 

SLTs do not accurately reflect the coagulation status of patients with ESLD. Platelet *number* does not correlate with platelet *function*, and fibrinogen levels do not accurately reflect structurally normal fibrinogen or dysfibrinogenemia (compounds with impaired fibrinogen polymerization) that are significantly increased in ESLD [55,56]. Another significant limitation of SLTs is the time necessary to complete the analysis. While the turnaround time for VET is 7–10 min, the turnaround time for SLTs depends on local facilities and can take between 60 and 90 min [57,58].

**Figure 2:**
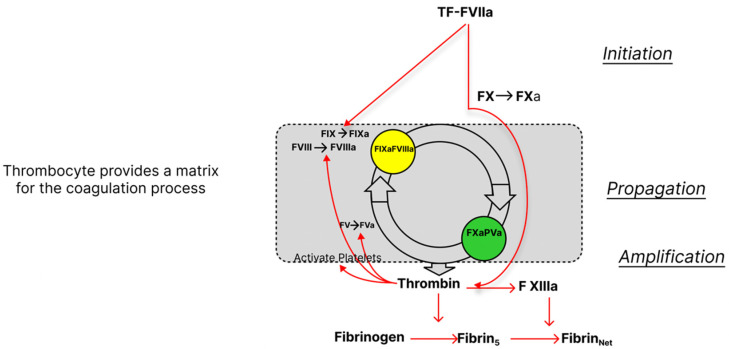
Cell-based coagulation process. The waterfall cascade hemostasis model was replaced in 2001 with a cell-based model [59]. The coagulation process begins with the release of tissue factor from endothelial cells, monocytes, and macrophages. Tissue factor then starts the initiation phase with F VIIa. Propagation and amplification follow, ending with a thrombin burst. The fibrin net is then built and stabilized with F XIII.

**Figure 3:**
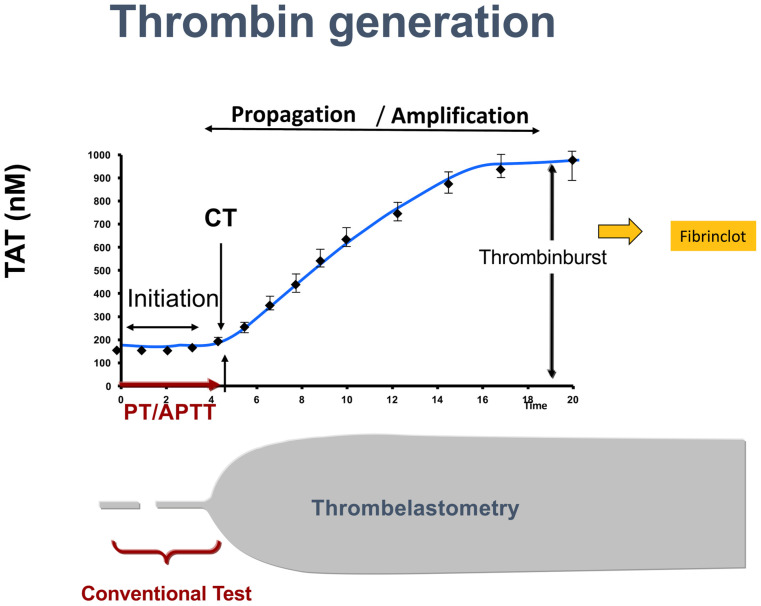
Standard laboratory tests assess only 5–10% of the coagulation process, while viscoelastic tests, like Rotem, assess the whole coagulation process. TAT: thrombin-anti-thrombin. PT: prothrombin time. aPTT: activated partial thromboplastin time.

In patients with ESLD, SLTs only provide a limited reflection of the current coagulation concept. Since 2001, a cell-based instead of a cascade-based coagulation model has been accepted as the standard concept [59]. According to the cell-based system, coagulation is divided into three phases: activation, propagation, and amplification. The activation phase begins with tissue factor release from a variety of cells which interacts with FVIIa resulting in the subsequent activation of FIX, FX, and prothrombin (FII). Prothrombin release is followed by fibrin production. In the propagation phase, prothrombin activates platelets that are used as a matrix where all metabolic processes can then occur. This is followed by activation of FV and FVIII to FVIIIa, F IXa, and F Va/Xa which leads to further release of thrombin. In the amplification phase, the release of thrombin is repeated as a positive feedback mechanism until thrombin achieves a 1000-fold higher concentration compared to baseline (thrombin burst). After the thrombin burst, a fibrin clot is formed (Figure 1). SLTs (such as PT and aPTT) assess only the initiation phase but not the other phases of coagulation and cannot give a complete picture of the status of coagulation (Figure 2 and Figure 3). 

One of the first studies which challenged the use of SLTs for predicting bleeding was published in 1981. Ewe et al. attempted to determine if bleeding time was dependent on platelet number and prothrombin time (PT) during a laparoscopic procedure in 200 cirrhotic patients having a liver biopsy performed with a 1.8 mm Meninghi needle [60]. The authors found no correlation between bleeding time and PT or platelet number. Two patients had a bleeding time of >12 min (mean 4 min 37 s) although their SLTs’ hemostatic values (PT and platelet count) were normal. 

One of the most important papers contributing to our understanding of coagulation in patients with ESLD was published by Tripodi et al. [1]. The authors evaluated endogenous thrombin generation in cirrhotic patients and healthy volunteers. The level of thrombin generation in healthy volunteers was significantly higher compared to cirrhotic patients. This difference, however, disappeared when TM was added to the assay. When thrombin binds to the TM receptor, protein C is activated. This then inhibits Factor Va and Factor VIIIa. These data have demonstrated that in cirrhotic patients, both pro- and anticoagulants are reduced.

SLTs are not designed to assess levels of anticoagulants. This is the main reason these tests are able to show a potential predisposition to coagulopathy, but not a hypercoagulable condition. Haas et al. performed a meta-analysis regarding using SLTs to predict bleeding events [61]. The authors were able to identify 11 guidelines and 64 studies evaluating the use of SLTs to predict bleeding. There were only three prospective studies with a total of only 108 patients included with no clear results. The authors concluded that SLTs were unsuitable for this purpose.

In contrast to SLTs, VET can overcome most of these disadvantages. In comparison to SLTs, VET dynamically assesses hemostasis in whole blood, not only in plasma, and includes the interaction between procoagulents, anticoagulants, and platelets. VET assesses the entire coagulation process to identify which part is disrupted and which allows for targeted therapy. Dr. Kang (working in Pittsburgh at this time) was the first to report the advantages of VET for the perioperative management of LT [62]. In 1985, he reported that transfusion requirements were reduced by 33% if VET was used. In a randomized controlled trial (RCT), De Pietrie et al. evaluated cirrhotic patients who underwent invasive procedures. Sixty patients were 1:1 randomized to guide hemostasis management based on VET or SLTs [63]. Patients in the VET group received significantly fewer RBC, FFP, and platelet transfusions compared to the SLT group. Post-intervention hemoglobin in the VET group was considerably higher compared to the SLT group. Bleeding events were not significantly different between groups. Similar results were reported by Kumar et al. [64]. These authors were able to demonstrate that VET-guided hemostasis management in cirrhotic patients with non-variceal bleeding could significantly reduce the number of transfused blood products without increasing the incidence of bleeding events. Similar findings were shown in children who underwent invasive procedures where VET-guided hemostasis management was associated with a significantly reduced rate of blood product transfusion [65]. However, VETs require careful interpretation. He et al. found that VET demonstrated normal coagulation status in stable cirrhotic patients and that hypercoagulability was not necessarily associated with the development of PVT [66]. Interestingly, in a meta-analysis performed one year later, the same group demonstrated the superiority of VET compared to SLTs in monitoring coagulation in patients with ESLD [67].

Currently, two VET devices are available: TEG and ROTEM. Hemostasis management with these two systems was assessed by Wikkelsø et al. in a Cochrane Database Systematic Review [68]. They enrolled 17 studies with a total of 1493 patients primarily having cardiac surgical procedures. Their analysis demonstrated reduced mortality (3.9% vs. 7.4%, RR = 0.52, 95% CI: 0.28–0.95) and significantly reduced transfusion rate of any blood product (red blood cells (RBC), fresh frozen plasma (FFP), or platelets) when TEG or ROTEM were used.

Karkouti et al. performed an RCT on patients having cardiac surgery and demonstrated a reduced transfusion requirement when VET was used [69]. Serraino et al. compared SLTs and VET in 8737 patients undergoing cardiac surgery and demonstrated a decreased need for RBC and platelet transfusion. However, no difference in mortality, prevalence of stroke, time on a ventilator, hospital stay, or incidence of bleeding could be demonstrated [70]. This report sparked a discussion among experts. In this study, the relative risk for mortality was 0.55 with a 95% CI of 0.28–1.1. If, however, the number of patients had been larger, differences in mortality would have been significant [71]. It was also emphasized that reducing the rate of RBC and platelet transfusion was associated with a significant decrease in acute renal failure (RR = 0.42, 95% CI 0.2–0.86) and correlated with a better outcome [72].

Although the advantages of VET were already shown in 1985 and several evaluations and meta-analyses have since demonstrated the beneficial effects of VET in reducing blood product transfusion as well as decreasing morbidity and mortality, many LT centers are still reluctant to use of VET and rely on SLTs.

## 4. Hemostasis in Cirrhotic Patients with Sepsis

### 4.1. Immune Dysfunction and Increased Risk of Severe Infection in Cirrhotic Patients

Patients with cirrhosis present with a persistent systemic inflammatory state and with different degrees of both innate and acquired immune dysfunction that is influenced by the severity of the liver disease. Portal hypertension, increased permeability of the intestine, and altered gut microbiota lead to intestinal bacteria translocation, endotoxemia, and worsening of systemic inflammation, which is the typical pathophysiology of severe infections in patients with cirrhosis [73,74,75]. It has been previously demonstrated that sepsis is diagnosed in one third of hospitalized cirrhotic patients [76].

### 4.2. Coagulation Changes in Cirrhotic Patients with Severe Infection and Sepsis

Coagulation disturbances in septic patients with cirrhosis are the result of coagulation activation induced by sepsis superimposed on an already altered hemostasis due to hepatic dysfunction. These changes in coagulation are dependent on the severity of both liver disease and infection and are not adequately evaluated by SLTs. VET and thrombin generation assay are global tests that can better reflect the hemostatic changes induced due to sepsis or severe infection in patients with cirrhosis.

Unfortunately, there are not many studies assessing coagulation changes in septic cirrhotic patients, and some of these were published more than 20 years ago [77,78,79]. Considering the heterogeneity of patients with liver disease and concomitant severe infections, sepsis, or septic shock, it is difficult to have a complete picture of the hemostatic system in this patient population.

### 4.3. Assessment of Hemostatic Changes Induced due to Infection in Patients with Compensated and Decompensated Liver Disease and Acute-on-Chronic Liver Failure

In patients without liver disease, severe infections result in coagulation activation mainly through the extrinsic pathway, with the inhibition of natural anticoagulants and hypofibrinolysis [80,81].

Severe infections occurring in patients with compensated liver disease affect their hemostatic balance, which often results in bleeding or thrombotic complications. In patients with decompensated cirrhosis, severe infections usually lead to a deterioration in SLTs, platelet count, and parameters of VET indicating hypocoagulability [77,79,82]. Resolving infection is associated with an improvement in coagulopathy, while in patients continuing to deteriorate, hemostasis worsens [77]. In a study using thrombelastography, viscoelastic parameters reflecting clotting initiation and propagation (r, k, alpha) were more useful than SLTs in identifying significant hemostatic changes in patients with persistent infection [77]. This reinforces the fact that VET is more sensitive for coagulation assessment in cirrhotic patients. When compared to cirrhotic patients without infections, or with mild infections, cirrhotic patients with sepsis have lower levels of pro- and anticoagulant factors, lower platelet counts, and increased soluble fibrin, independent of the severity of the liver disease [83]. These findings suggest that inflammation-induced coagulation activation leads to the consumption of coagulation factors and platelets in cirrhotic patients with severe infections or sepsis [83].

Sepsis causes 25–30% of acute-on-chronic liver failure (ACLF). About 50% of patients with ACLF from other causes will develop sepsis in the course of the disease [84]. A more recent study has demonstrated that patients with ACLF presenting with an already decompensated hemostatic system have further aggravation of coagulopathy by sepsis [82]. When compared to ACLF in patients without sepsis, patients with sepsis have been shown to have worse SLTs, but the differences were not statistically significant [82]. When compared to non-septic patients with ACLF presenting with hypocoagulability (confirmed by VET), patients with sepsis had more severely disturbed hemostasis manifested by longer clotting times and lower clot amplitude on thrombelastography [82]. The measurements of individual coagulation factors demonstrate that patients with ACLF have decreased levels of protein C and AT III, as well as increased levels of vWF, FVIII, and tissue factor activity. All these parameters worsened significantly in patients who developed sepsis, and this deterioration correlated with increased mortality at 28 days [82].

A recent study comparing patients with decompensated cirrhosis with and without infections revealed decreased levels of natural anticoagulants (protein C, S and, ATIII), FVII, and prolonged INR in infected patients, which normalized with infection resolution [85]. However, in this study, the decrease in anticoagulants was not correlated with a higher degree of thrombin generation, which was similar in infected and non-infected patients [85].

In non-cirrhotic patients, sepsis leads to defects in platelet aggregation [86]. Similarly, patients with decompensated cirrhosis and bacterial infections present with decreased whole-blood platelet aggregation when compared to cirrhotic patients without infections [85]. This could be explained by the increased release of NO and prostacyclin induced by bacterial endotoxins in infected patients with cirrhosis, contributing to further impairment of primary hemostasis [73,87]. As opposed to secondary hemostasis, which improved after infection resolution, a further impairment of platelet aggregation was noted in cirrhotic patients that recovered from infection independently of baseline platelet count and agonist used [85].

It is known that severe infections and sepsis in patients without liver cirrhosis are usually accompanied by hypofibrinolysis. Infected patients with decompensated cirrhosis present mixed fibrinolytic changes, with lower plasminogen levels, higher levels of tissue plasminogen activator, and similar levels of plasmin–antiplasmin complexes compared to non-infected patients [85].

### 4.4. Endothelial Damage and Release of Endogenous Heparinoids with Severe Infection

Bacterial infection is associated with a significant heparin-like effect in chronic liver disease, impairing coagulation due to the release of endogenous heparinoids from endothelial surfaces, and possibly due to activation of mast cells with subsequent release of heparin and other mediators [78,79]. In their study, Montalto et al. demonstrated the presence of a heparin-like effect due to endogenous heparinoids using heparanase-modified thrombelastography in cirrhotic patients with severe infections, but not in non-infected cirrhotic patients or in infected patients without cirrhosis [79]. This heparin-like effect disappeared after the resolution of the infection [79]. These results were confirmed by Zambruni et al. who were able to demonstrate higher anti-Xa levels in infected compared to non-infected cirrhotic patients [78]. Endotoxin or cytokines are involved in the release of heparinoids from endothelial surfaces in a dose-dependent manner. This may be the reason this effect was not shown in non-infected cirrhotic patients (with lower endotoxin levels). In non-cirrhotic patients with infections, this effect was not demonstrated because, with unaltered liver function, the clearance of heparinoids is not affected [79]. These findings are important for explaining the increased bleeding risk in cirrhotic patients with severe infections.

### 4.5. Disseminated Intravascular Coagulation in Cirrhotic Patients

Sepsis is the most common cause of disseminated intravascular coagulation (DIC). In DIC, both coagulation factors and platelet counts are decreased mainly due to consumption. This, in combination with increased fibrinolytic activity, represents the routine diagnostic criteria for DIC [88,89]. Diagnosing DIC in the presence of chronic liver disease is challenging due to the similarities in coagulation tests with both conditions. The problem of diagnosing DIC becomes even more complicated in patients with decompensated cirrhosis and ACLF. In these cases, DIC is often over-diagnosed with the usual DIC scoring systems. It is not surprising, then, that the diagnosis of DIC is often missed in patients with sepsis and ACLF, as the coagulation markers in ACLF patients can be severely altered even before they develop an infection.

In conclusion, patients with ESLD are prone to hypercoagulability on the level of primary, secondary, and tertiary hemostasis. Despite decreases in both coagulation and anticoagulation factors in the liver, these patients have an imbalanced system prone to both bleeding and clotting. ED and increased thrombin generation play a central role in hypercoagulability associated with ESLD. SLTs do not accurately reflect the coagulation profile of patients with ESLD. VET is more reliable than SLTs for managing coagulation in this patient population. VET is particularly helpful in patients with ESLD complicated due to sepsis, a situation with very complex coagulation disturbances.

## Data Availability

Not applicable.
